# Teaching and Learning of Piano Timbre Through Teacher–Student Interactions in Lessons

**DOI:** 10.3389/fpsyg.2021.576056

**Published:** 2021-06-10

**Authors:** Shen Li, Renee Timmers

**Affiliations:** ^1^Department of Psychology, Central China Normal University, Wuhan, China; ^2^Department of Music, University of Sheffield, Sheffield, United Kingdom

**Keywords:** piano teaching, piano timbre, teacher-student interaction, tone production, mind-body integration

## Abstract

The ability to play the piano with a variety of timbres requires a performer to have advanced pianistic skills. Little is known about how these skills are acquired and developed in piano lessons and what the role is of elements such as concepts, technique, sonic outcomes, and bodily movements. To investigate the teaching and learning of piano timbre, the lessons of three pairs of university-level teachers and students (two teachers and three students) were observed, during which they behaved as usual in the first two lessons and were asked to use a dialogic teaching approach in the third lesson. Verbal communications of teachers and students about timbre were coded and analyzed, aiming to gain insight into the teaching/learning process of piano timbre and the roles of embodiment and teacher–student interaction in the context of higher music education. The results suggest that piano timbre is not learned through imitation or as “fixed” and objective knowledge, but as a co-constructed conception between the teachers and the students. The meaning of timbre goals in piano lessons is enacted through “in-the-moment” bodily experience and embodied through performance actions. This study contributes to the understanding of piano timbre as a multifaceted phenomenon and illustrates the teacher's role in developing the student's mind–body integration involved in tone production.

## Introduction

Pianists are often convinced that their “sound” is distinct from others even if they play the same musical piece on the same instrument (Valière et al., 2019). However, scientists and musicians hold conflicting views about whether piano timbre can be varied by applying different qualities of touch without changing other performance parameters (i.e., touch–tone relationship^1^) (for a recent review, see Goebl et al., 2014). This paper will consider this debate in the context of piano lessons when students learn to play a piece of music with a variety of timbres^2^ under the instruction of piano teachers.

The talking that happens in a lesson plays an important role in achieving a shared understanding between teachers and students. Analyzing such verbal interaction, Woody (2000) reported more frequent use of feeling-oriented descriptors in lessons rather than musical instructions (i.e., literal descriptions of pitch, rhythm, volume, etc.) or technical instructions, while according to Colprit (2000), instrumental music teachers frequently talk about teaching targets in terms of concrete musical results (i.e., achieving a certain tone) rather than physical behavior (e.g., the motion of the bow). Burwell (2006) further compared the differences between instrumental lessons and vocal lessons and found that the use of metaphorical language occurred more frequently in the context of vocal teaching. What verbal descriptions and techniques are used may depend on what is worked on in lessons. In this study, we will examine the role of different types of verbal descriptors related to musical, cognitive, and physical domains when teachers and students work on piano timbre in lessons.

This study is interested in answering the following research questions: What does piano timbre refer to in a piano lesson? How do teachers and students interact to acquire and develop timbre? What is the role of each type of verbal descriptor (musical, cognitive, and physical) in the communication of piano timbre? Three piano lessons of three pairs of university-level teachers and students were observed to address these questions (nine in total). The first lesson was open in focus. In the second, teachers were encouraged to work on piano timbre, while in the third lesson, teachers were asked to use a dialogic approach to work on timbre. Before presenting the details, a theoretical framework is offered that discusses touch–tone relationships in piano performance and the roles of gestures, bodily awareness, and proprioceptive feelings in piano performance and teaching. It closes with a consideration of teacher–student interaction during instrumental teaching.

## Theoretical Framework

### The Touch–Tone Relationship: A Highly Contested Topic

The debate surrounding the touch–tone relationship in piano performance has been going on for over a century. Acousticians advocated that piano timbre can hardly be varied while keeping the performed intensity unchanged because piano timbre is only determined by the force of the finger on the key (Parncutt and McPherson, 2002) and the final hammer velocity (Bryan, 1913), which directly relate to intensity. One degree of force produces one degree of intensity and, hence, only one quality (Ortmann, 1925), and pianists need to rely on the parameters of intensity and time to vary piano timbre (Seashore, 1937; Turner, 1939). On the other hand, pianists train themselves for decades to develop sophisticated touch qualities that vary in depth of key-press, finger/hand shapes, rigidity, and movement directions (see discussion below). The acoustical perspective of timbre production seems to have challenged pianists' views on the control of piano timbre. Do pianists tend to neglect scientific findings on tone production, continuing to pay more attention to more artistic views and manners? The answer is, apparently, no, as world-class pianist Rosen (2002) clarified:

*Inside the piano, the elaborate arrangement of joints and springs will only cause the hammer to hit the strings with greater or lesser force. The graceful or dramatic movements of the arms and wrists of the performer are simply a form of choreography that has no practical effect on the mechanism of the instrument, although if it looks more graceful, it may sound more exquisite, not only to the public but to the pianist convinced by his own gestures* (p. 24).

Gát (1974) commented that, even though the experiments conducted on the piano touch–tone relationship may not be incorrect, they cannot be accepted as the only truth, since piano playing cannot only be explained rationally. American pianist C. G. Hamilton explained the physical mechanism of tone production on the piano (Hamilton, 2012): “…the hammer is thus left to fly the rest of the way to the string, actuated by the momentum already imparted to it…it means that the really effective part of the hammer stroke is actually without the control of the player” (p. 31).

The above pianists' statements on tone production demonstrate that pianists are not “blind” to the scientific views on the touch–tone relationship; instead, they clearly understand the mechanical limitations of the production of piano timbre. Nevertheless, researchers need to understand the perception of piano timbre from a broader and holistic perspective, and consider the following questions: Why do pianists prefer to say that they have changed the timbre rather than the intensity of performance? Why do students and teachers repeatedly work on different touch types in piano lessons and experiment with them focusing on one piano tone or chord, even though this would seem to be pointless from the acousticians' viewpoint? It seems that the value, meaning, conceptualization, and significance of timbre in a musical context is different from the areas that acousticians normally focus on, and this has left a rich space for this research to explore.

Therefore, the notion of piano timbre in the present paper is not focused on an investigation of spectral evidence (i.e., *timbre itself*); instead, it will explore the beliefs of teachers and students about piano timbre (i.e., *timbre perception*) and present empirical data related to the ways in which timbre is mobilized and talked about in teaching situations. Given the restricted possibilities for variations in sound color on a piano, the notion of timbre in this study will embrace attributes such as articulation, intensity, and melody, which typically are not included in the definition of timbre, but is related to pianists' own accounts of piano timbre because they blur together in the perception of piano tones (Li and Timmers, 2020). Moreover, the performing body (cf. Dogantan-Dack, 2011) and multisensory perception are seen as crucial components in understanding the perception of piano timbre. For example, the haptic–tactile feedback of a keystroke influences a pianist's perception of piano timbre as different touch types (pressed vs. struck) generate differences in feeling, in addition to sound (i.e., finger-key noise and key-keyframe noise, Goebl et al., 2005, 2014); while listeners' perceptions of piano timbre may be influenced by the visual perception of a musical performance (Parncutt, 2013; Li, 2020).

In short, although it remains contested to what extent pianists have independent control over piano timbre as a spectral measurement, to do justice to the conceptualization of piano timbre among pianists, educators, and learners, research will need to adopt a broader and more inclusive approach that considers body and sound, including the role of movements, proprioception, and cross-sensory imaginations. Moreover, touch and movements are not merely motor skills in piano playing but are embodiments of interpretative ideas and auditory expectations, as we will discuss next.

### Piano Timbre and Gestures

Research on movements in music performance generally makes a distinction between sound-producing and sound-facilitating gestures (Jensenius et al., 2010). Piano touch and its different forms are generally categorized as sound-producing gestures. MacRitchie (2015) conducted a systematic review of piano touch and relevant biomechanical knowledge to piano performance. Her research considered individual differences (e.g., hand anthropometry, difference in training, etc.) in the choice and utilization of touch types and suggested an effective and scientific way of achieving an expressive musical performance while minimizing the risk of injury. Berman (2002) summarized several crucial aspects of piano touch, including: weight (how much weight is applied to the key), mass (how much of the body is involved in the keypress), speed (of the keypress), perception of depth (comparison of deep or shallow touch), shape of the fingers (curvature, amount, and location of contact on the pad or fingertip), and *in* or *out*: “in” (pouring weight into each note) and “out” (pulling the fingers away from the keys, “… as if grabbingthe sound from the keyboard and bringing it out”) (p. 6). These studies imply the importance of utilizing different touch forms for pianists, even though the effect on piano timbre is limited.

Little is known about the function of sound-facilitating gestures in shaping pianists' own experiences of piano timbre. The control of the body is crucial in piano performance, including bodily tension and relaxation, the use of weight and force, and finger–arm coordination. In relation to the coordination of different parts of the body, Godøy and his colleagues (Godøy, 2006; Godøy et al., 2010) highlight that body movements during a musical performance co-articulate in similar ways as phonemic gestures in continuous speech (Hardcastle and Hewlett, 2006). The motion of coarticulated gestures has characteristics of temporal development, in the sense that both past events and future events influence present events: the positions and shapes of effectors are affected by recent actions, while being part of, and shaped by, the preparation for future actions (Godøy, 2013).

The literature reviewed above suggests that the study of piano gestures, and by implication of piano timbre, should adopt a holistic perspective by switching the attention from specific touch qualities to a global unit of performative gestures that include both sound-producing and sound-facilitating gestures. The tone production process should be regarded as unfolding at multiple levels as when the movement of one effector (e.g., the finger striking the piano or the hand creating a chord) “spills over” into neighboring parts of the body (Godøy et al., 2010). Therefore, it is possible to assume that the corporeal experiences that are associated with piano timbre perception extend from a singular effector (e.g., finger) to the entire body of the performer. Indeed, it is feasible that the direction, posture, weight, effort, and tension of movements of the entire body are involved in performers' experience and production of piano timbre. These close associations between timbre perception and corporeality have become research interests for several researchers in recent years (e.g., Prem and Parncutt, 2008; Dogantan-Dack, 2011; Li and Timmers, 2020).

### Proprioceptive Feelings and Body Awareness in Piano Playing

The study of gestures and body movement is relatively prominent in empirical research of piano performance. Less attention has been paid to the role of proprioceptive feelings and bodily awareness.

#### Proprioceptive Sensations

The need for a student to have proprioceptive sensations in piano playing has been clearly indicated in the views of several piano pedagogues. In 1927, Thomas Fielden, a professor of piano at the Royal College of Music, emphasized the idea of sensing muscular contraction in addition to his other key emphasis on perfect timing, in successful tone production. He suggested that a student who did not sense muscular contraction when playing should place a hand lightly on a table and then press with enough tension to experience the feeling of muscular contraction. At around the same time, the pianist, Levinskaya (1930) also suggested that it was imperative for pianists to be aware of which lever (i.e., joint) they intended to use and then to create a firm ground for operating the action by fixing some joints with muscular contraction. In this way, a sense of proprioception helps the pianist to monitor and improve their playing action.

In addition to the benefit of improving motoric skills, proprioceptive information also functions as a sign of conscious awareness while undertaking musical activities. For the purpose of this study, we will limit the notion of consciousness to a sense of body awareness and mental focus when discussing the process of tone production (e.g., Godøy, 2011). Piano teachers often tell their students to “play with your mind, not just your fingers!” This state of playing consciously as the combination of physical movement and mental awareness can be facilitated and achieved through using proprioceptive information. By feeling body sensations, the student can integrate their mind (intentions) with their body and create a concentrated mental state of “playing while thinking.” As claimed by Acitores (2011), proprioception theory can be seen as an embodied account of musical consciousness, in which the body works as the basis for consciousness. The teaching of piano tone production can be seen, therefore, as the process of sharing (knowledge of) proprioceptive information with the student, specifically regarding the sensory experience associated with performance actions. Evidence of this can be found in the verbal instructions from teachers such as: “It's too tense.” or “Too much weight.” Although the teacher does not experience any tension or weight themselves at that moment, they can infer the sensory experience from hearing the sound quality produced by the student. This is consistent with the “mirror neuron” theory, that to understand and perceive a sound is to internally simulate the movement related to that sound (Leman and Godøy, 2010).

#### Body Awareness

Body awareness relates to the subjective experience of proprioceptive and interoceptive sensations, associated with enhanced notice of and differentiation between bodily sensations, cognitions, and emotions (Mehling et al., 2011). Approaches based on T'ai Chi and Alexander technique have been reported to be widely used in piano playing and pedagogy contexts to enhance performers' body awareness. Pianist David (1996) shares her experience of how a shift in body awareness has had a positive effect on muscular injury:

Awareness of the sensation is in fact the only way to dismiss boredom and convert muscular pain into pleasure… Immediately, the arms round up more gracefully, the hands feel strong without tensing up, the fingers feel graceful, the wrist follows the forearm, the legs consolidate one's stance, the body runs into a silent piano (p. 22–23).

She also recommended that pianists ought to learn to discern body sensations and discomforts (e.g., a stiff neck and an awkward posture) and turn them into actions (e.g., to relax, stretch out, and lighten the shoulder and neck). These strategies align with therapeutic approaches such as breath awareness, repetition and training, refinement of noticing, discriminating, and discerning physical sensations (Mehling et al., 2011). The ideas of David (1996) imply that body awareness brings changes in actions, which further mediate and modify a performer's perceptions, intentions, and emotions during piano playing. In this sense, the continuous loop between actions, body sensations, and mindfulness emphasizes the unity of body and mind.

### Teacher–Student Interactions

The teachers and students who participated in this study are university level, and students received individual lessons from a master performer. This model, sometimes referred to as master–apprentice, has been simplified as the pairing of a dominating teacher and a receiving student, where the dominant mode of student's acquisition of musical skills is to adopt or imitate (Jørgensen, 2000). Critics of this model highlight limitations in active participation of the student, leading to limited levels of independence (Nielsen and Kvale, 1997; Jørgensen, 2000; Schiavio et al., 2018). For example, according to Schiavio et al. (2018), this teaching process can be characterized as involving a unidirectional stream where information and knowledge is transmitted from the teacher to the student, which generates an asymmetrical relationship, and may lead to an overly self-conscious and stressful situation for both participants. The characteristics of one-to-one tuition in the context of higher education has been investigated in the studies of Gaunt (2009, 2011), which highlight the intensity of the teacher–student relationship and the power of the teacher over the student's learning outcomes. While students were generally positive about this one-to-one relationship, they were also fearful about what might happen if the relationship faltered (Gaunt, 2009). Therefore, Lehmann et al. (2007) suggested that the master–apprentice model may gradually develop into a mentor–friend model, in which context the dominant role of the teacher is decreased by the increasing the contribution and participation of the student, resulting in a strong sense of student autonomy. In our study, we will be observing teacher–student interactions in a Chinese university context, which is often criticized by western scholars in terms of heavy work on pianistic skills and rote learning (Davidson, 1989), teacher-centered approach (Kuzmich, 1995; Wong, 2002), and more student playing while less talking (Benson and Fung, 2005).

To enhance students' independent learning, the adoption of a dialogic teaching approach (Alexander, 2008) has been advocated with adoption trials showing positive results for music performance development (Meissner, 2017, 2021; Meissner and Timmers, 2020). Dialogic teaching has also been related to the notion of scaffolding, in terms of constructing shared conceptions (Alexander, 2000), and scaffolding students' active participation (Muhonen et al., 2016). Alexander (2010) described the approach of dialogic teaching as “harnessing the power of talk to stimulate and extend pupils' thinking and advance their learning and understanding. It helps the teacher to diagnose pupils' needs more precisely, to frame their learning tasks and assess their progress” (p. 1). As mentioned by Alexander (2010, 2018), dialogic teaching not only aims to enhance students' communication skills in the class by the power of talk, but it also influences the way that teachers and students conceive of knowledge. Hereby, we understand dialogic teaching as asking *open questions* and using *dialogue* rather than teacher presentation (Alexander, 2008, 2010; Meissner and Timmers, 2020). We used dialogic teaching as a suggested approach in the third week to enhance the students' active involvement in learning.

Zorzal and Lorenzo (2019) suggested that physical contact between the teacher and student works as a platform from which the teaching of the essential haptic contact required to play a musical instrument can take place, to meet students' proprioceptive needs (e.g., body posture, muscle relaxation, and use of the fingers). Simones et al. (2015) found that teachers adjusted the gestural scaffolding approaches according to a student's skill level: for example, conducting gestures were commonly used for higher-level students; while mimicking gestures (i.e., to imitate sound-producing intentions) were more usually adopted when working with students at lower levels to demonstrate action-related knowledge. With respect to our study on piano timbre, we will assume that non-verbal communications can be helpfully employed to support a student's understanding of timbre goals, whether this is accompanied by verbal explanation, modeling, or otherwise.

This paper is also concerned with utilizing cognitive theories to explain the teacher–student interaction, in line with current trends of embodied and enactive approaches (Elliott and Silverman, 2014; Van Der Schyff, 2015) to the study of music education and music cognition. As mentioned, several scholars have been critical toward traditional forms of teaching, in which teacher–student interaction risks to be too unidirectional (Van Der Schyff, 2015; Schiavio et al., 2020). A traditional format may furthermore emphasize teaching as primarily cognitive information transmission (see for a discussion, Van Der Schyff et al., 2016): e.g., through verbal instruction or aural modeling given by the teacher; so that a task-specific, mental representation is constructed followed by rule-based generative processes, which guide subsequent performance plans and actions (Schiavio et al., 2020). Van Der Schyff et al. (2016) criticized this model as promoting a form of musical development that leads to fixed, objective knowledge stored “in the head.” The study of Schiavio et al. (2020) indicated that the learning outcomes of novices were better in turn-taking mode (when participant–participant or participant–computer played sections in turns) than the imitation mode (when participant solos or duos copied what a computer played), and argued that this is due to active (co-) participation in the generation of musical materials, implying that musical learning is best conceived and most successful as a shared, in-the-moment, musical experience. In contrast to some of these critical stances, we will see in this study (to pre-empt some of the results) co-production happening also within a traditional master–apprentice teaching format.

## Methdology

### Study Design

A teaching observation study was conducted to gain insight into the process of teaching and learning piano timbre in a semi-naturalistic context. It aimed to investigate what teachers do to enhance the students' learning of piano timbre using linguistic and non-verbal communication strategies, and to investigate what types of concepts and methods are used. Three piano lessons of three pairs of teachers and students were video recorded (3 × 3 = 9 lessons). There was a 1-week interval between each lesson. In the first lesson, teachers were asked to teach the students as they would normally do; in the second and third week, teachers were asked to work specifically on timbre as part of the lesson. To promote verbal communication about piano timbre and active contributions from the student, a dialogic teaching approach was suggested to be used in the final week. A list of sample questions was provided (see Appendix) to the teachers, to help the student to think about timbre and become more aware of the possibility of changing timbre, so teacher and student can develop their understanding of timbre together. The step-by-step approach can help the teachers and students relieve the psychological discomfort caused by observation and reduce the resistance to the researcher in terms of the interference of teaching content and teaching style. Therefore, they may feel more at ease to work as usual in week 1 in the face of cameras, then adjust teaching and learning goals in week 2, and try the new teaching style (using more open questions and dialogues) in week 3.

Three teacher–student pairs from the Music Department of Henan University (China) took part in the teaching observation study. This involved a female teacher (age = 45; teaching years = 20) with her student S1 (female; age = 21) (pair A) and a male teacher (age = 56; teaching years = 30) with two of his students S2 (female; age = 20) and S3 (male; age = 17) (pairs B and C). They both expressed their interest and agreed to take part in the study. An information sheet about the study was provided in advance of the video recordings of the lessons, and written consent was obtained from all participants. All participants were aware that the observation aims were relevant to the teaching and learning of piano timbre, before the recording of the lessons. Apart from that, the teachers were asked to teach the same musical piece and progressively work on it over the course of 3 weeks. The students were required to practice the music in advance up to a high level.

### Data Analysis

The nine recorded lessons lasted around 7.5 h in total. Data analysis addresses what the notion of piano timbre refers to in piano lessons (timbre goal), how teachers and students interacted to achieve these timbre goals (time allocation and learning behaviors), and which verbal descriptors (different types) were used in the communication of timbre concepts.

#### Timbre Goals

Data analysis focused on the events when the teacher and student explicitly communicated about piano timbre^3^. We used the notion of “rehearsal frame” (Duke, 1994; Colprit, 2000; Küpers et al., 2014) to identify an event of timbre teaching/learning, which starts from the moment a teacher identifies an aspect of the student's performance that needs to be improved regarding timbre, and stops at the moment when the specific goal is accomplished or changed to a new goal. ELAN (2020)^4^ was used to process the video recording, which is a platform that enables one to mark the starting/ending points of an event with precision and control. In the end, there were 77 video excerpts of teaching and learning of timbre for data analysis, which totaled around 8,742 s (2 h, 25 min, 42 s). The conversations in these video excerpts were accurately transcribed for further qualitative data analysis.

#### Time Allocation and Learning Behaviors

The data analysis of the time allocation was concerned with three types of teacher/student behaviors, i.e., teacher modeling (T-modeling) and teacher–student talking (TS-talking), and student playing (S-playing)^5^, which is based on the standard paradigm (verbal and non-verbal behaviors) used in the analysis in the studies of teaching observations (e.g., Kelly, 2003; Zhukov, 2012). The performance sessions and talking sessions were easy to discriminate using the Sonic Visualiser (2020)^6^. The time devoted to TS-talking was not split into parts of teacher talking and student talking due to students talking less (< 9% of the total amount of words in the video transcription). As a further development of observing students' learning, we analyzed the frequency of the students' learning behaviors following Zhukov's study (2012) and differentiated behavior into the following categories: playing, answering, questioning, agreeing with teacher, and making excuses for their poor performances.

#### Types of Verbal Descriptor

Verbal content analysis was used (Mayring, 2004) to analyze the conversations between the teacher and student. The first step was to allocate text interpretation deductively into existing categories derived from previous research and theory (Mayring, 2004). Developing from previous teaching observation studies on instrumental lessons (Colprit, 2000; Woody, 2000; Burwell, 2006), this study found that the participants' verbalization can be analyzed in terms of how they are mapped onto the musical domain (e.g., concrete sound attributes), cognitive domain (e.g., felt emotion, metaphors), or physical domain (e.g., actions, movements). The coding of musical/physical/cognitive domain descriptors was conducted using the NVivo software (Welsh, 2002). This software was used to generate the frequency of appearance. Each domain can be described as follows.

- Physical domain: descriptions of the use of the body, arm, and fingers, as well as the energy, velocity, force, and movement type. This is based on Colprit (2000) who discriminated between descriptors of physical behaviors (e.g., motion of the bow, fingering, spacing, position, etc.) and musical descriptions.
- Musical domain: descriptions that are associated with musical parameters, such as musical tempo, dynamics, articulation, sustain or soft pedal, timing, and musical phrase or structure. As mentioned, Colprit (2000) referred to this domain as “musical descriptions,” while Burwell (2006) labeled it as “literal vocabularies,” which are used to address issues of pitch, volume, and structure.
- Cognitive domain: descriptions that are relevant to emotions, metaphors, and images, as well as body awareness and expressive intentions. Woody (2000) used the terminology “feeling-oriented” in his paper to label felt emotions and moods. Burwell (2006) referred to “metaphorical vocabularies” when referring to experiential, emotional, and figurative meanings. We integrated these two categories and complemented them with descriptions that include body awareness and sensations (Acitores, 2011; Mehling et al., 2011) and expressive intentions (e.g., expressive, deadpan, exaggerated, and projected) (see, e.g., Davidson, 2005).

The procedure of data analysis started quantitatively from the identification of timbre events after familiarization with the whole data set (i.e., “time allocation”). Next, the qualitative analysis was conducted from two perspectives: (1) deductive coding of TS-talking into physical, musical, and cognitive domain; (2) inductive coding that aimed to capture closely what was worked on in the teaching excerpt (timbre goal). For this, the information of each video excerpt was summarized with a label (e.g., “think timbre as a man's voice,” “timbre and hand coordination,” “timbre of the last note,” “horn-like timbre,” etc.). Through comparison and categorization of the labels, the timbre-related events were grouped into higher-level categories (as further explained in the *Results* section).

## Results

### Timbre Goals

Two types of timbre goals stood out from the whole data set (77 timbre-related events), namely, metaphorical timbre (30) and ideal timbre (23). The remaining timbre-related events each contributed only small portions of the data and were removed from the analysis of the data.

#### Metaphorical Timbre

This timbre goal was described by the teachers using vivid visual/aural images, such as a “male/female voice” and “shimmering river” (Pair A), “Brazilian dancing” and a “bell-like sound” (Pair B), and “Lute timbre” on a piano (Pair C). These metaphors were found to be used consistently by the teachers over 3 weeks and illustrate the rich imagery employed in verbal communication (examples in Table 1 below). Interestingly, there are several adjective descriptors commonly used by all three pairs of teachers and students, for instance, bright, dark, rich, heavy, and open. In addition, metaphorical timbre seemed inseparable from emotional expression, for example, T1 explained a relationship between affections of struggling, doubting, and needing, and the imitation of a male/female voice on the piano; T2 associated the metaphor of Brazilian dancing with emotions of feeling free, open, and enthusiastic.

**Table 1:** Musical excerpts and quotes of the teachers using Metaphorical Timbre.

**Teacher-Student pair**	**Timbre goals**	**Bar and Score information and Performed pieces**	**Quotes**
Pair A	*Princess and the earl*	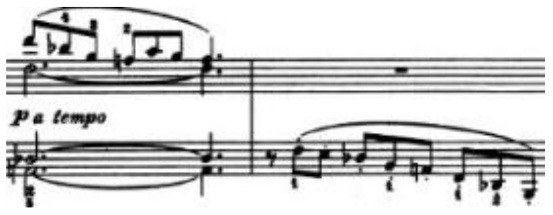 Bar 10–11 *(“May” from The Seasons*, Tchaikovsky, 1997)	“These two phrases are a conversation between the *princess and the earl*; These two have different timbres–you should express clearly” (week 1) “You can interpret as *men's voice*. Not only crescendo, but much *thicker”* (week 2)
Pair C	*Lute timbre on the piano*	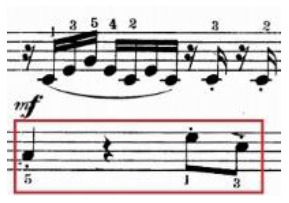 (whole piece–left hand) *(Small Preludes and Fughettas, No. 1, C minor*, Bach, 1986)	“It is like *the sound of a lute*; it is also composed to imitate the lute, so the left hand must be *staccato*.” (week 1) “Your hands are too loose. Actions of strike and release should be very rapid.” (Week 3)

#### Ideal Timbre

This type is closely associated with an aesthetic dimension in the evaluation of tone quality, where teachers used language referring to: “goodness/badness,” “appropriateness,” “beauty,” and “satisfaction.” It is noteworthy that the two teachers often used expressions of “THE sound” or “THIS sound” to refer to ideal timbre. The teachers, therefore, have certain criteria of “what is good or bad” and “what is right or wrong” and encouraged the student to follow these values. With the teacher's appraisal and feedback, the students learned to avoid producing wrong/bad/undesired timbres.

### Time Allocation

The duration of each lesson ranged from 37 to 57 min (Mean = 49′36″, SD = 7′53″). The amount of time spent on the teaching and learning of timbre varied across pairs and weeks. Table 2 displays the length of timbre events (labeled as “timbre length”), as well as the percentage of teacher modeling (T modeling), student playing (S-playing), and teacher–student talking (TS-talking) happening in each piano lesson. These data indicate that the verbal and non-verbal behaviors of three teacher/student pairs are balanced in the first 2 weeks, but for all three pairs, the TS-talking increased in the third week (right column in Table 2) due to the use of dialogic teaching.

**Table 2:** The duration spent on the teaching/learning of timbre in a piano lesson across 3 weeks in three pairs and the percentage of time of T-modeling, TS-talking, S-playing in a those piano timbre excerpts.

	**Week 1**				**Week 2**				**Week 3**			
	**Timbre length**	**T-Modeling %**	**TS-talking %**	**S-playing %**	**Timbre length**	**T-Modeling %**	**TS-talking %**	**S-playing %**	**Timbre length**	**T-Modeling %**	**TS-talking %**	**S-Playing %**
PAIR A	18′19″	32.9%	31.7%	35.4%	9′38″	38.9%	35.8%	25.3%	10′39″	24.3%	49.1%	26.6%
PAIR B	20′58″	37.45	28.2%	34.3%	22′00″	39.2%	33.2%	27.7%	20′08″	39.5%	45.0%	15.6%
PAIR C	6′16″	41.0%	45.0%	14.1%	20′44″	37.5%	60.5%	1.9%	22′13″	16.2%	70.3%	13.52%

The students' learning behaviors were coded and counted (see Table 3): the largest category was S-playing, and the amount of S-talking (answering, agreeing, questioning, and excusing) only took < 9% of the total amount of words in transcript. Table 3 displays the frequency of each type of S-talking per lesson. The results indicated that the students' verbal contributions increased by weeks, especially an increase in asking questions and answering. The three students did behave differently: student A seemed to dislike verbal communication, student B was more active in asking questions and answering, while student C showed more agreement on the teacher's feedback (e.g., “yes, it's too loud”). In terms of the questioning behavior, the students either asked specific questions [e.g., “is it (timbre change) about the contact size of the fingertips?” —student C] or general questions (e.g., “I don't know how to vary the sound”—student B). In response, the teachers developed the communication by actively listening and scaffolding the students' learning by explaining, demonstrating, or clarifying.

**Table 3:** The frequency of the students' verbal behaviors in the learning of piano timbre.

**Pairs**	**Behaviors**	**Week 1**	**Week 2**	**Week 3**
Pair A	Questioning	0	0	0
	Answering	0	0	1
	Agreeing	0	0	0
	Excusing	0	0	0
Pair B	Questioning	3	2	**5**
	Answering	1	0	**4**
	Agreeing	1	6	1
	Excusing	2	2	2
Pair C	Questioning	0	0	**2**
	Answering	2	3	**5**
	Agreeing	0	2	0
	Excusing	0	0	1
SUM		**9**	**15**	**21**

*Bold values indicate the number of questioning and answering increased in week 3*.

### Verbalization of Piano Timbre

The physical domain descriptors (151) occurred with the highest frequency in the teachers' and students' verbalization of piano timbre, followed by the musical domain descriptors (82) and cognitive domain descriptors (78). In the *physical domain descriptors*, the most frequently occurring subcategories were energy (44), actions (41), and speed (19). Action-related descriptions specify the kinesthetic–motional features of sound-producing gestures (direction, duration, speed, and weight) and are taken from everyday life movements (e.g., pulling/pushing, brushing, and beating) but using hands as articulators of sounds on the piano (see Li and Timmers, 2020). The teaching process relied heavily on the teacher's demonstration of both the “right” and “wrong” types of physical action. Together with an aural example, this gave the student a continuous and contrasting experience of touch and its impact on timbre. Table 4 illustrates several examples of action-type descriptors accompanied by either superimposed images or arrows showing the trajectory of movement(s).

**Table 4:** Examples of physical actions and the corresponding explanation in words and movement.

**Physical actions**	**Verbal explanation**	**Images**
挑*tiao* (pulling)	Using one finger to touch the key in readiness, like “pulling a string with nails” together with a rising hand. (T1)	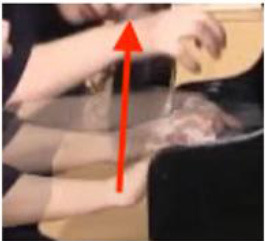
提*ti* (lifting)	Similar to *tiao*, but with an emphasis on lifting the wrist. (T1)	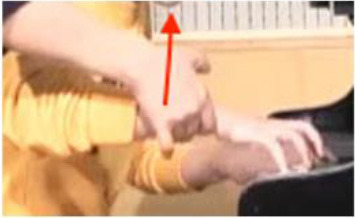
勾*gou (hooking)*	The end of the finger is acting like a hook with an upward movement. The touch is very short and crisp but elastic, where the wrist keeps almost still. (T2)	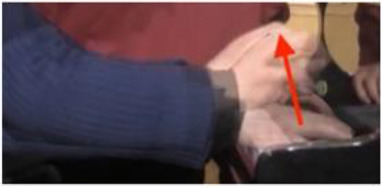
	Wrong action demonstration by the teacher: In the playing of a quick passage of octave chords, it is wrong to use too much wrist moving up and down, which caused “heavy” and “dead” timbre. *	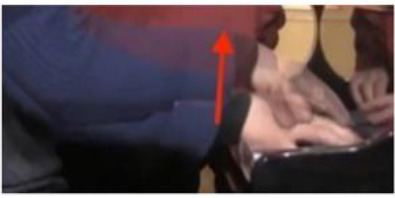
拂*fu (brushing)*	To brush. Use of very flat finger rather than curved finger to gently touch the key. This is used in the playing of darker timbre. (T2)	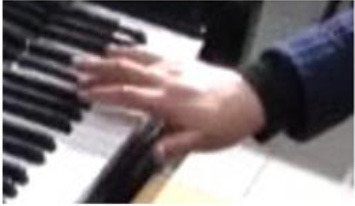
	In opposite with the preceding item “fu,” the use of a very curved but not angular finger helps to make a brighter timbre.	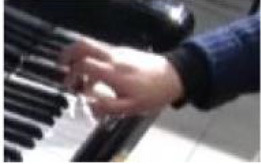
抓*zhua (grasping)*	A rapid sliding motion on the keyboard, like “grasping movement,” using a quick and short touch inwards. This is used to produce an elastic timbre. (T2)	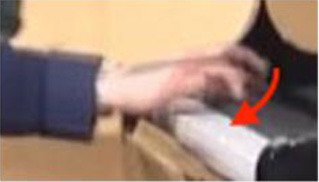
推*tui (pushing)*	Use of a lower wrist to push the weight into the keyboard. (T2)	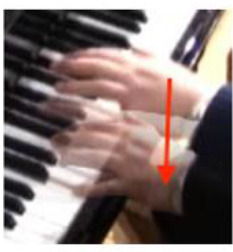
拍pai* (beating)	Use of very quick and jerky movement. Striking the piano, like “beating,” up and down to the piano. * (T1)	
抽chou* shrinking)	This is similar to *Pai*, but with a jerky movement inwards. * (T1)	

The actions marked with ^*^ were less preferred by the teachers who recommended to avoid using these actions in piano playing.

The most frequently occurring descriptor from the *musical domain* was that of volume (34), which was considerably higher than articulation (12), tempo (8), tone duration (4), and phrasing (4). The teachers raised questions to students in terms of timbre–intensity differences and demonstrated to the students how to obtain timbre variety while keeping intensity the same in order to raise their awareness of mobilizing various timbres and touch in performance. For example:

Pair C T: The homework I gave you last week, is to think how to improve the timbre.
S: Yes, I wanted to make a crescendo from here.
T: How about the touch (to change the timbre)? Don't just think of making contrasting dynamics, that's just one aspect. Different techniques will bring different timbres, different timbres represent beauty in various ways. How do you make a more beautiful sound? *Harmony, dynamics, are just part of it.*

References to a metaphorical idea (24), an emotional feeling (20), body awareness and sensations (14), and expressiveness (11) were the four most common approaches in the *cognitive domain* descriptors. Using a metaphorical approach, the physical aspect of timbre production was taught by drawing parallels with everyday movements. “Playing is like walking” was a frequently occurring comparison among the three pairs, where the parallel was made between finger movement and leg movement to metaphorically explain the influence of speed, continuity, weight, and height on timbre. Vocabulary relating to emotions and feelings was used frequently by the two teachers, such as pleasant, awe, desire, mysterious, sad, calm, and depressing. The vocabulary used to describe musical expressiveness included contrasting, exaggerating, non-expressive, plain, deadpan, and straightforward. The teachers in this study used these words to instruct their students to vary the degree of expression. In the codes of body awareness and sensations, the teachers actively guided the student's attention to either sounds or the body and helped the student to discern the feelings of discomfort, tension, and weight.

### Dialogic Teaching

In the final week (third lesson of a student), the two teachers in this study were encouraged to use dialogue when working with the student on piano timbre. The male teacher preferred to use dialogue (*N* = 20 in total; 4 with student B; 16 with student C) more than the female teacher (*N* = 5, with student A) in the communication of timbre goals. Nevertheless, all pairs used more open questions in the last week. Quotes from Pair C are selected below (interpretation from the authors' perspective is offered in square brackets).

Example 1:

T2: …Have a listen this time, what do think is different compared to last time [T starts with an open question].
S3: It's deeper…[S's response]
T2: [Hmm…] Did it change? What do you think changed? [T rephrases the question and wants more explanation from the student].
S3: Well, this time I felt the sound was more “solid”—compared with the last performance—which was “crisp”—this one was more “solid,” and “heavier” [S explains the difference in more detail].
T2: The reason for the so-called heavier sound was because you played heavily. [T explains the technical reason for what caused a “heavier” sound]. But you should—and maybe you're not—feel the energy flowing from the back of the hand and going through the fingers [T tries to guide S to think of the use of energy].
S3: Yes, I felt that.

The teacher's questioning had a positive influence on the student's independent thinking—the student was encouraged to discriminate differences in piano timbre and used metaphorical descriptors to conceptualize their experiences, including “deeper,” “solid,” and “heavier.” The teacher complemented this with a reference to proprioceptive feelings, to let the student feel differences in timbre by distinguishing “where the energy comes from.”

### Non-verbal Scaffolding

**Figure 1:**
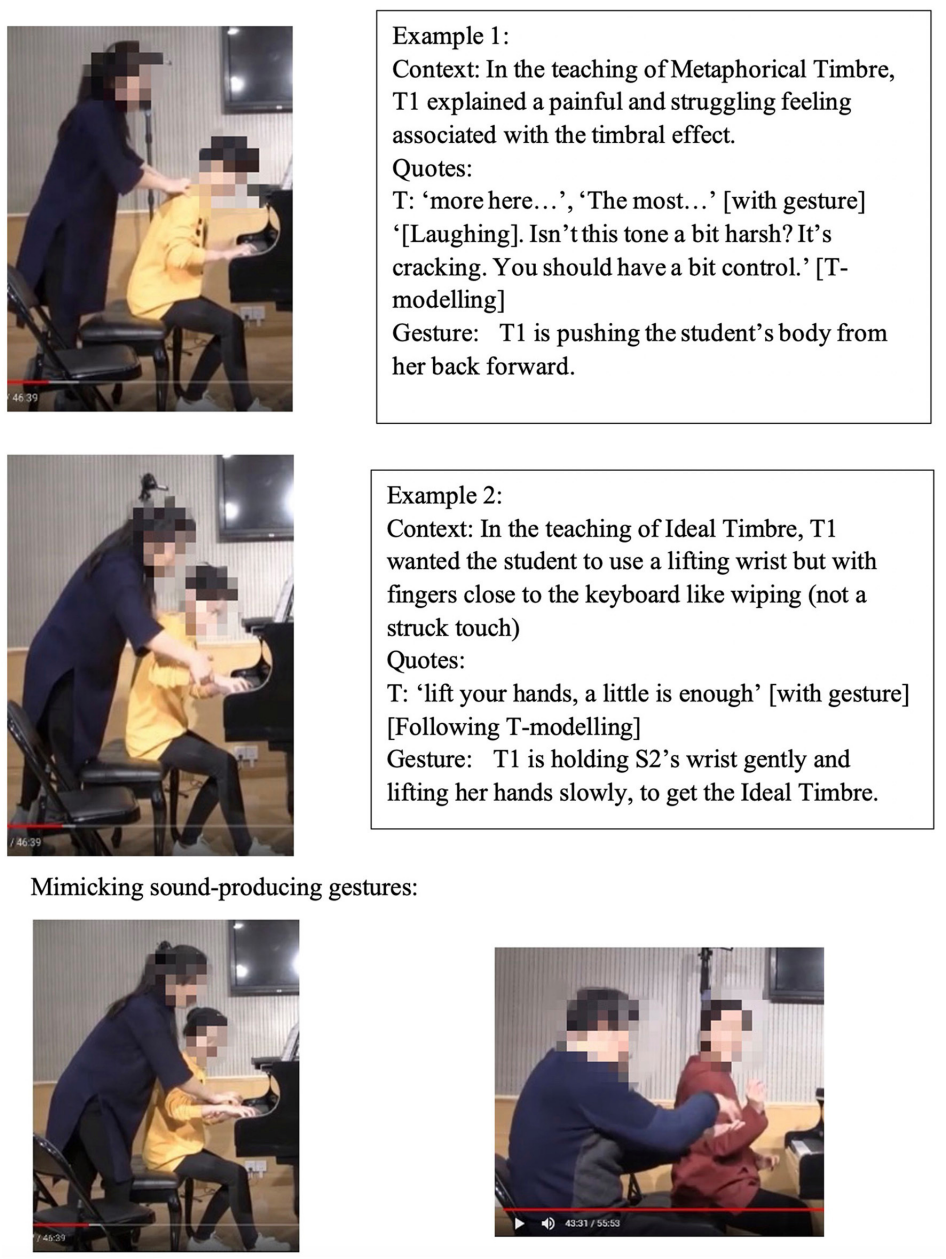
Non-verbal behaviors in the observation study related to metaphorical timbre, ideal timbre, and illustrating sound-producing gestures.

It was interesting to note that in some circumstances, the teaching and learning of timbre targets was realized neither through words nor sounds, but through non-verbal behaviors such as direct or indirect physical touch and the teacher's mimicking gestures. This study did not systematically calculate the frequency and types of non-verbal behaviors as previous studies did (Zhukov, 2012; Simones et al., 2015). However, as an exploratory study with the focus on piano timbre, some interesting examples have been selected (Figure 1) to explain how non-verbal behaviors worked together with verbal behaviors.

The two examples in Figure 1 identify a difference between the two teachers in the mimicking of sound-producing gestures—the female teacher directly touches her student to mimic the sound-producing action, while the male teacher mimics on his own hand. This could potentially be because the student is of the opposite gender with the male teacher, or he considers it inappropriate to use touch irrespective of gender, or for a different reason. Additionally, the female teacher uses touch as a main form of explanation; in Example 1, T2 did not give an explicit explanation of what is referred to as “more,” but she clarified her meaning by pushing the student from the back, which also changed the performance posture. It seems the expression “more” represents a mixture of more expressiveness, more energy, and more bodily movement.

## Discussion

### “In the Same Boat”—A “Student-Centered” Teaching and Learning Process

The first research question investigated in this study is the conceptualization of piano timbre among teachers and students and what timbre refers to in piano lessons. We found ideal timbres and metaphorical timbres to be the two largest clusters of timbre goals. The category of ideal timbres is related to the quality dimension of piano timbre, for instance, a subjective reaction and unified experience of piano tones (Ortmann, 1935) that may involve a person's subjective and aesthetic judgments (e.g., “good tone,” Neuhaus, 1993). The cluster of metaphorical timbres is in accordance with previous verbalization studies on timbre (Bellemare and Traube, 2005).

Subsequently, we examined how the teachers and students interact to achieve timbre goals with sounds, gestures, and verbal communications. In the teaching and learning of ideal timbres, the teachers frequently mentioned right/wrong and appropriate/inappropriate touch and wanted their student to “copy” their gestures and sounds to acquire musical skills and to avoid possible injuries. However, this process is not unidirectional, as the teachers had to listen actively to the students' performance, diagnose their troubles and needs, and guide their attention to experience either visual, auditory, or kinesthetic feedback. Such an “in-the-moment” and shared musical experience may lead to a reciprocal interaction between the teacher and the student (De Jaegher and Di Paolo, 2007; Schiavio et al., 2020), leading to a “student-centered” teaching process. The teachers also needed to understand the gestures and actions of the students to experience the body sensations in the student's experience of the tone production process. As Hyry-Beihammer (2010) argued, the teacher is “in the same boat with the student” rather than taking a master's “authoritative role,” specifically if combined with an open approach toward input from the student. Through the intervention of dialogic teaching approach in week 3, all three students employed more verbal interactions with their teachers, responded with more questions and answers instead of agreeing and excusing. However, S-playing is still the biggest category of the students' learning behaviors, far exceeding the proportion of S-talking, which is consistent with Benson and Fung's (2005) findings. Open questions and dialogue can be used to stimulate students' thinking about musical interpretation and enhance active participation (Meissner, 2021), thus, increasing the possibility of “co-produced” conceptions of piano timbre.

In the teaching and learning of metaphorical timbres, the teachers used several metaphors and imageries consistently in the 3 weeks, and there seemed to be a consensus between the teacher and the student as both did not need re-explanations the next time the same metaphor/imagery was used. In this sense, metaphors can be regarded as scaffolds that facilitate the students' learning of performance gestures. Along with the teachers' repeated coaching and the student's continuous practicing of performative gestures, these linguistic scaffolds may fade into the background (“coaching-fading” mode, Byrne, 2005), together with a stronger feeling of “autonomy” or ownership in the student's learning process (Meissner and Timmers, 2020). Therefore, the meaning of an abstract metaphor or image such as a “lute-like” or “horn-like” timbre can be said to have been *enacted* using the body and performance development (De Jaegher and Di Paolo, 2007), rather than existing as fixed, objective knowledge that is transmitted from the teacher to the student on the basis of a “correspondence” schema.

### The Investigation of Language: Meaning Construction of a Timbre Goal

The last research question examined in this study was, “What is the role of each type of verbal descriptor in the communication of timbre concepts?” We examined the descriptions of timbre as related to a musical, cognitive, or physical domain. The results revealed that timbre goals were more often described using physicality than concrete musical results or metaphors, images, and emotions. This leads to the impression that the focus of piano teaching is mainly on techniques (Karlsson and Juslin, 2008). However, we believe that different types of verbal descriptions interact to collaboratively construct the meaning of a timbre goal.

Given that timbre goals may seem to be abstract (e.g., metaphorical descriptions) and subjective (criteria of “right, good, and appropriate”), the extensive use of *physical domain* descriptions may play an important role in providing a physical basis and embodied experience for the understanding of the timbre concepts. Along with the student's active experimentation with sounds and gestures, there is a transmission from bodily knowledge to musical knowledge by incorporating sensorimotor skills with intentions. Dalcroze's approach applied in the settings of music teaching reveals similar insights to this study, using concrete bodily movement to enrich musical understanding (Juntunen and Hyvönen, 2004). Clearly, the sonic outcomes of timbre goals can be partly demonstrated by teacher modeling, but the *musical domain* descriptions (e.g., “It's getting louder here”) can also help the student to make sense of the concurrent changes in other performance parameters resulting from the variation of timbre, hence, enabling them to generate an explicit performance plan (Woody, 1999). This can effectively project an aural image onto the student's short-term memory and guide their normal practice (Hallam, 1998; Meissner and Timmers, 2020). Consistent with previous studies (Barten, 1998; Woody, 2000; Burwell, 2006), figurative language and instructions on both perceived and felt emotions occurred frequently in *cognitive domain* descriptions, but they were found inseparable from the descriptions of corporeality. For example, the teacher's instruction of a “painful” expression in Pair A was fulfilled through proprioception of muscular tension in the student. These results imply that musical experience is a cross-domain experience where sound and imagery interconnect, and pianists use knowledge from one domain of experience (physical movements) to structure another domain of experience (musical notes), creating a blended space (Zbikowski, 2002).

Previous research has already indicated that humans lack precise vocabularies to describe timbre impressions (Fales, 2002; Wallmark and Kendall, 2018; Saitis and Weinzierl, 2019), and rely on everyday analogies and experience to describe timbre, such as visual shapes (Adeli et al., 2014), visual texture (Giannakis, 2006), and smells (Crisinel and Spence, 2010, 2012). The vocabulary used by the teachers and students were sometimes flowery and vague, especially in the cases of metaphorical timbre, which heavily rely on the use of adjectives, imageries, and analogies; nevertheless, it reveals the use of timbre-related cross-modal correspondences (Adeli et al., 2014). The teachers' demonstration may be clearer and more intuitive than verbal instructions, but it seemed that the use of language and concepts in combination with performance technique and sounds is key to creating an in-depth understanding and exploration of timbre.

This study highlights the importance of language in the communication of timbre goals between university-level teachers and students, which is distinct from some previous studies, which indicated that standalone modeling can be effective in music teaching (Rosenthal, 1984; Sang, 1987; Woody, 2006). The factor of the student's age influences the amount of teacher talking; as the study of Meissner and Timmers (2020) mentioned, the teaching of young children's musical expressiveness faces the difficulties of “turning verbal teaching into actions.” The student participants in this study are university-level students, but still face the challenges of understanding the teachers' verbal instructions about a metaphorical timbre (e.g., darker timbre was interpreted as playing slower by the student in Pair B, but this was rejected by the teacher). However, this study would suggest that teachers and students take advantage of conversation and dialogue in piano lessons. Timbre goals have left an open space for both the teacher and the student to explore, in which the intentionality and meaning can be co-constructed and, within which, language plays an important role in terms of conceptualizing and identifying performative gestures, timbres, perceived/felt emotions, body sensations, and imageries.

### “Get Beyond the Notes”—A Multimodal and Embodied Approach to Teaching and Learning Timbre Concepts

This study indicates that the teachers let their students feel piano timbre by using multiple senses, not just hearing. In line with previous studies (Simones et al., 2015; Zorzal and Lorenzo, 2019), this study found that the non-verbal behaviors of the teachers meet the technical and proprioceptive needs of the students in the focus on timbre by demonstrating the right sound-producing gestures and adjusting the students' performance gestures and posture, so that the students can fine tune from where and in what way weight is applied, with what speed and lightness to move, and learn what haptic experiences are required to play the piano.

Modeling was used along with verbal instructions in this study; in addition, the understanding of timbre targets came from the contributions of other sensory modalities such as physical touch, gestural visualization, metaphorical imagery, and proprioceptive feelings. All these elements helped to create a shared, multidimensional space for both the teachers and students to understand and explore the possibility of performed sounds—therefore, there is a need to “get beyond the notes” (Davidson, 1989; Barten, 1998). Timbre is no longer a “hardwired” sonic feature of a musical piece, but is deeply rooted in bodily experience and feelings, which, in turn, enrich the conception of piano timbre for both the teacher and the student so bringing more expressive qualities to timbre perception.

The teachers helped the students to continuously refine their performative gestures, where sound is the ultimate goal. This forms a perception–action cycle (Godøy, 2011): the body movements and sensations contribute to the experiences of the tone production process; meanwhile, performance actions and gestures are adjusted and driven by an auditory anticipation (e.g., a desired tone quality or a “richer” tone). In this action–perception loop, the teachers actively guided the students' attention toward sound, intentionality, gestures, or body sensation, to help them to connect movements, sensations, and concepts. An embodied perspective on musical learning seems justified: that motor skills related to timbre production are gained through sound discovery in the interaction with the piano (Schiavio et al., 2017).

## Conclusion

This study found that the teachers preferred to name timbre goals in metaphorical terms (e.g., men's voice, brighter sound, etc.) or as an ideal type (e.g., right timbre). However, examination of the verbalization showed that these timbre goals were explained more frequently using descriptions of physicality, instead of concrete music results, emotions, and imageries, implying that the meaning of timbre goals is enacted through the real-time bodily experience and embodied performance gestures. Dialogue and non-verbal gestures contributed to create a shared conception of timbre goals between the teachers and the students, where proprioceptive knowledge played an important role, more so than teacher modeling. These results have challenged the ideas of “teacher-centered” and verbal–cognitive teaching process and suggested the adoption of an in the moment and multimodal perspective in the teaching practice of tone production. These are useful proofs of concepts of embodied and enactive accounts of piano education and mind–body integration in tone production.

This study only observed three teacher/student pairs within an HE context. Interesting results may arise when comparing a variety of teachers, for instance, teachers and students of various levels of experience regarding the amount of time devoted to timbre and types of verbal instruction (e.g., Goolsby, 1997). Previous studies have suggested that cross-cultural differences may exist in the metaphorical descriptions of performance skills in relation to piano timbre (Zhao, 2007) and teacher–student interactions (Davidson, 1989). Furthermore, work can also be developed in the field of cross-cultural comparison (e.g., Benson and Fung, 2005; Bonastre et al., 2017) to compare the differences in the conceptions and approaches between music teachers from different cultures.

## Data Availability Statement

The datasets presented in this article are not readily available because they consist of non-anonymized video recordings. Requests to access the datasets should be directed to the authors.

## Ethics Statement

The studies involving human participants were reviewed and approved by University of Sheffield Ethics Reviewers. The participants provided their written informed consent to participate in this study. Written informed consent was obtained from the individual(s) for the publication of any potentially identifiable images or data included in this article.

## Author Contributions

Both authors were involved in the design of the research. SL was responsible for data collection, data analysis, and the first draft of this manuscript. RT helped to restructure the theoretical framework, refine the arguments, and make the manuscript more readable and clear. Both authors contributed to the article and approved the submitted version.

## Conflict of Interest

The authors declare that the research was conducted in the absence of any commercial or financial relationships that could be construed as a potential conflict of interest.

## Footnotes

^1^It is phrased as “the mystery of touch and tone” by Ortmann (1925).

^2^Note that we will use the term “piano timbre” throughout the text as an overarching term to refer to the perception of timbre in the context of piano performance, rather than “tone quality” or “tone color.” These seem to be used interchangeably with “timbre” in the writings of several pianists (Kochevitsky, 1967; Berman, 2002; Hamilton, 2012) even though there might be reasons to separate the terms (see Łetowski, 1992). The present paper will use “timbre” to maintain consistency with previous studies that also investigated the perception and production of timbral nuances produced within one instrument (e.g., Traube, 2004; Bellemare and Traube, 2005; Holmes, 2011). In addition, this study will examine metaphors and vocabularies of participants, encompassing concepts that go beyond what is understood by “tone quality” (cf. Saitis and Weinzierl, 2019).

^3^Due to the diversity of verbalization used to communicate about piano timbre and also because of some cultural differences, the verbalization of piano timbre in the Chinese context may also be replaced with a few terms such as *yinzhi* (“tone quality”), *yinse*, (“tone color” or “timbre”), *shengyin*, (“sound”), or *yin*, (“the tone”).

^4^https://archive.mpi.nl/tla/elan

^5^They will appear as abbreviated afterward.

^6^https://www.sonicvisualiser.org/download.html
