# Supplementary material for: Teaching and Learning of Piano Timbre Through Teacher–Student Interactions in Lessons
Source: Front Psychol. 2021 Jun 10;12:576056. doi: 10.3389/fpsyg.2021.576056 (PMC8222694; doi:10.3389/fpsyg.2021.576056)
Supplement: Supplementary file 1 [file Table_1.DOCX]

**Appendix**

**Sample of open questions in the final lesson (teaching observation study)**

Please use more open questions in the final lesson. This might help your student to think about timbre (what to achieve and how to do this) and become more aware of the possibility to influence timbre. Here are some examples of open questions. Please choose to use where appropriate.

***Type A: Questions asked before playing:***

>Which part of the piece would you like to work on with timbre?

 >What timbre would you like to create?

 >How would you like to do this?

***Type B: Questions asked after playing:***

>Which part of the piece do you find difficult for working on timbre?

 >Why is it difficult? What are the problems?

***Type C: Reflections on the teacher’s performance or the student’s own performance:***

>What do you think of my timbre? Did you notice any differences between mine and yours?

>What do you think of your timbre (compared to last time)? Any changes or improvements?
